# Physiology of γ-aminobutyric acid production by *Akkermansia muciniphila*

**DOI:** 10.1128/aem.01121-23

**Published:** 2023-12-13

**Authors:** Prokopis Konstanti, Kate Ligthart, Christos Fryganas, Patinios Constantinos, Hauke Smidt, Willem M. de Vos, Clara Belzer

**Affiliations:** 1 Laboratory of Microbiology, Wageningen University & Research, Wageningen, the Netherlands; 2 Food Quality and Design, Wageningen University & Research, Wageningen, the Netherlands; 3 Human Microbiome Research Program, Faculty of Medicine, University of Helsinki, Helsinki, Finland; University of Illinois Urbana-Champaign, Urbana, Illinois, USA

**Keywords:** *Akkermansia muciniphila*, γ-aminobutyric acid, GABA, acid stress tolerance, gut microbiota, glutamate decarboxylase

## Abstract

Gut bacteria hold the potential to produce a broad range of metabolites that can modulate human functions, including molecules with neuroactive potential. One such molecule is γ-aminobutyric acid (GABA), the main inhibitory neurotransmitter of the central nervous system in animals. Metagenomic analyses suggest that the genomes of many gut bacteria encode glutamate decarboxylase (GAD), the enzyme that catalyzes GABA production. The genome of *Akkermansia muciniphila*, a mucin specialist and potential next-generation probiotic from the human gut, is predicted to encode GAD, suggesting a contributing role in GABA production in the human gut. In this study, *A. muciniphila* was grown in batch cultures with and without pH control. In both experiments, *A. muciniphila* was found to produce GABA as a response to acid (pH <5.5), although only when GABA precursors, either glutamate or glutamine, were present in the medium. Proteomic analysis comparing *A. muciniphila* grown with and without precursors at pH 4 did not show a difference in GAD expression, suggesting that it is expressed regardless of the presence of GABA precursors. To further investigate the function of *A. muciniphila* GAD, we heterologously expressed the *gad* gene (encoded by locus tag Amuc_0372) with a His tag in *Escherichia coli* and purified the GAD protein. Enzyme assays showed GAD activity in a pH range between 4 and 6, with the highest specific activity at pH 5 of 144 ± 16 µM GABA/min/mg. Overall, our results demonstrate the ability of *A. muciniphila* to produce GABA as an acid response and unravel the conditions under which GABA production in *A. muciniphila* occurs.

**IMPORTANCE:**

*Akkermansia muciniphila* is considered to be a beneficial bacterium from the human gut, but the exact mechanisms by which *A. muciniphila* influences its host are not yet fully understood. To this end, it is important to identify which metabolites are produced and consumed by *A. muciniphila* that may contribute to a healthy gut. In the present study, we demonstrate the ability of *A. muciniphila* to produce γ-aminobutyric acid (GABA) when grown in an acidic environment, which often occurs in the gut. GABA is the major inhibitory neurotransmitter in the central nervous system and is present in the human gut. For this reason, it is considered an important bacterial metabolite. Our finding that *A. muciniphila* produces GABA in acidic environments adds to the growing body of understanding of its relationship with host health and provides an explanation on how it can survive acid stress in the human gut.

## INTRODUCTION

Gut bacteria hold the potential to produce a broad range of metabolites that can modulate human functions, including molecules with neuroactive potential such as γ-aminobutyric acid (GABA). GABA is a non-proteinogenic amino acid that has diverse physiological functions and is produced by a wide range of organisms, from microorganisms to plants and animals (1). In humans, GABA is the principal inhibitory neurotransmitter of the central nervous system and plays a key role in brain function and the body’s response to stress (2). Besides the brain, significant amounts of GABA are also present in the human gut, but its role is poorly understood. Evidence so far supports that GABA influences gut motility and secretion, mucosal function, and inflammation (3).

Importantly, both humans and bacteria can produce GABA within the gut. Studies in germ-free mice showed that levels of fecal GABA were depleted compared to conventionally raised mice (4), indicating that bacteria are producing significant amounts of intestinal GABA in the gut (5, 6). Recent studies in humans showed that the concentration of GABA and glutamate in feces from the general population is associated with the microbial composition, further supporting the possibility of gut bacteria as a source for intestinal GABA (7).

**FIG 1:**
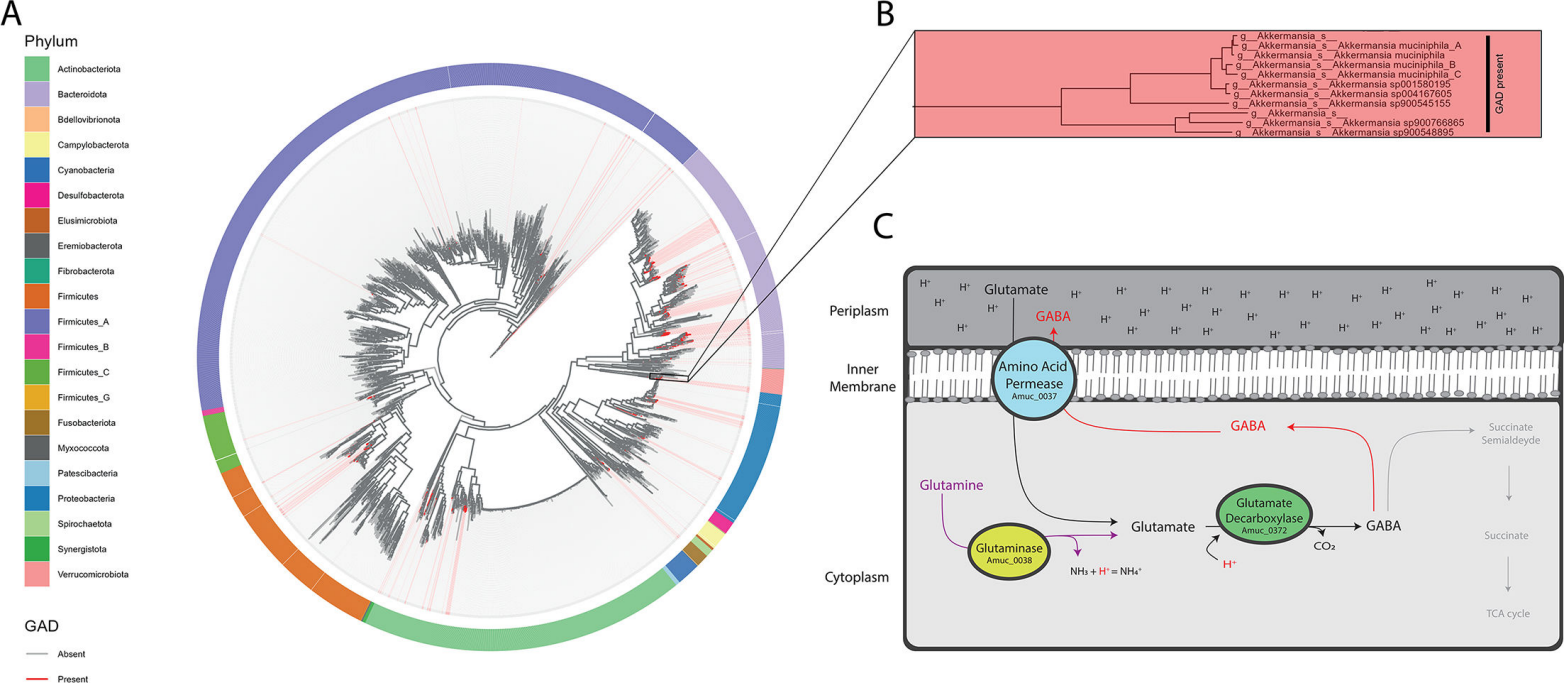
(**A**) Phylogenetic tree of the MGnify species catalog showing the presence (red) or absence (gray) of the glutamate decarboxylase (GAD) gene (15). (**B**) Zoomed in on *Akkermansia* spp. showing all tested *A. muciniphila* genomes harbor the GAD gene. (**C**) GABA production in bacteria, with the gene names from *A. muciniphila* and the pathway for converting GABA into succinate in gray. In an acidic environment, glutamate can be taken up by the bacterium through the amino acid permease ion-selective antiporter. Glutamate is converted by a glutamate decarboxylase using CO_2_ and another proton into GABA in the presence of pyridoxal 5′-phosphate. When glutamine is used as a precursor, an extra proton is consumed as it is converted to glutamate by a glutaminase. In cases like *A. muciniphila*, when a bacterium does not have the ability to convert GABA into succinate, GABA is not converted into succinate but rather exported into the environment by the amino acid permease.

*Akkermansia muciniphila* is one of the bacteria predicted to produce GABA (Fig. 1A and B) (8). It is a member of the core gut bacteria and a potential next-generation probiotic microbe that has been linked to metabolic and mental health (9–13). *A. muciniphila* is a mucin-degrading bacterium and the sole representative of the phylum Verrucomicrobia in the human gut. A lack or decreased abundance of *A. muciniphila* has been associated with a variety of diseases, such as metabolic and neurodegenerative disorders and inflammatory diseases (13, 14). *A. muciniphila* colonizes the mucus layer of the intestinal tract, and its physical proximity to the intestinal cells has led to various studies addressing host-microbe interactions. An important finding has been that *A. muciniphila* is capable of improving gut-barrier function in obesity and is linked with the gut-brain axis by increasing immune and metabolic signaling (11).

The synthesis of GABA in gut bacteria is catalyzed by the enzyme glutamate decarboxylase (GAD), which requires glutamate, CO_2_, and a proton to produce GABA in the presence of the cofactor pyridoxal 5′-phosphate (PLP) (16). Conversion of glutamate to GABA leads to the consumption of protons and has been proposed as one of the major acid resistance mechanisms in bacteria (Fig. 1C) (17). Next to GAD, two other proteins have been described for their importance in GABA production: an amino acid transporter and a glutaminase (Fig. 1C) (8). The amino acid permease is an antiporter responsible for transporting GABA and glutamate across the membrane (Fig. 1C) (17, 18). Glutaminase is essential when glutamine instead of glutamate is used as a substrate for GABA production. In that case, glutamine will first be deaminated by the glutaminase into glutamate, also consuming a proton, before being converted to GABA (17).

The GAD enzyme in bacteria has been extensively studied in pathogenic strains of *Escherichia coli* and *Listeria monocytogenes* and in non-pathogenic bacteria like *Lactoccoccus lactis*, *Limosilactobacillus* (prev. *Lactobacillus*) *reuteri*, and species from the genera *Bifidobacterium* and *Bacteroides* (6, 19). However, metagenomic analyses predicted that many other gut-colonizing bacteria possess the GAD gene, along with the genes for the amino acid transporter and glutaminase (6). Previous genomic analyses showed that *A. muciniphila* harbors all the relevant genes for GABA production, including Amuc_0372 (glutamate decarboxylase), Amuc_0037 (amino-acid permease), and Amuc_0038 (glutaminase), suggesting that *A. muciniphila* can produce GABA (8) (Fig. 1C). However, GABA production by *A. muciniphila* has so far not been experimentally validated. To address this, we studied the ability of *A. muciniphila* to produce GABA and to clarify the conditions under which GABA is produced by *A. muciniphila*.

## RESULTS

### *A. muciniphila* produces GABA as a response to acid stress in batch cultures

To explore the possibility that *A. muciniphila* produces GABA, we cultivated *A. muciniphila* in the presence or absence of GABA precursors, glutamate or glutamine, in batch cultures.

**FIG 2:**
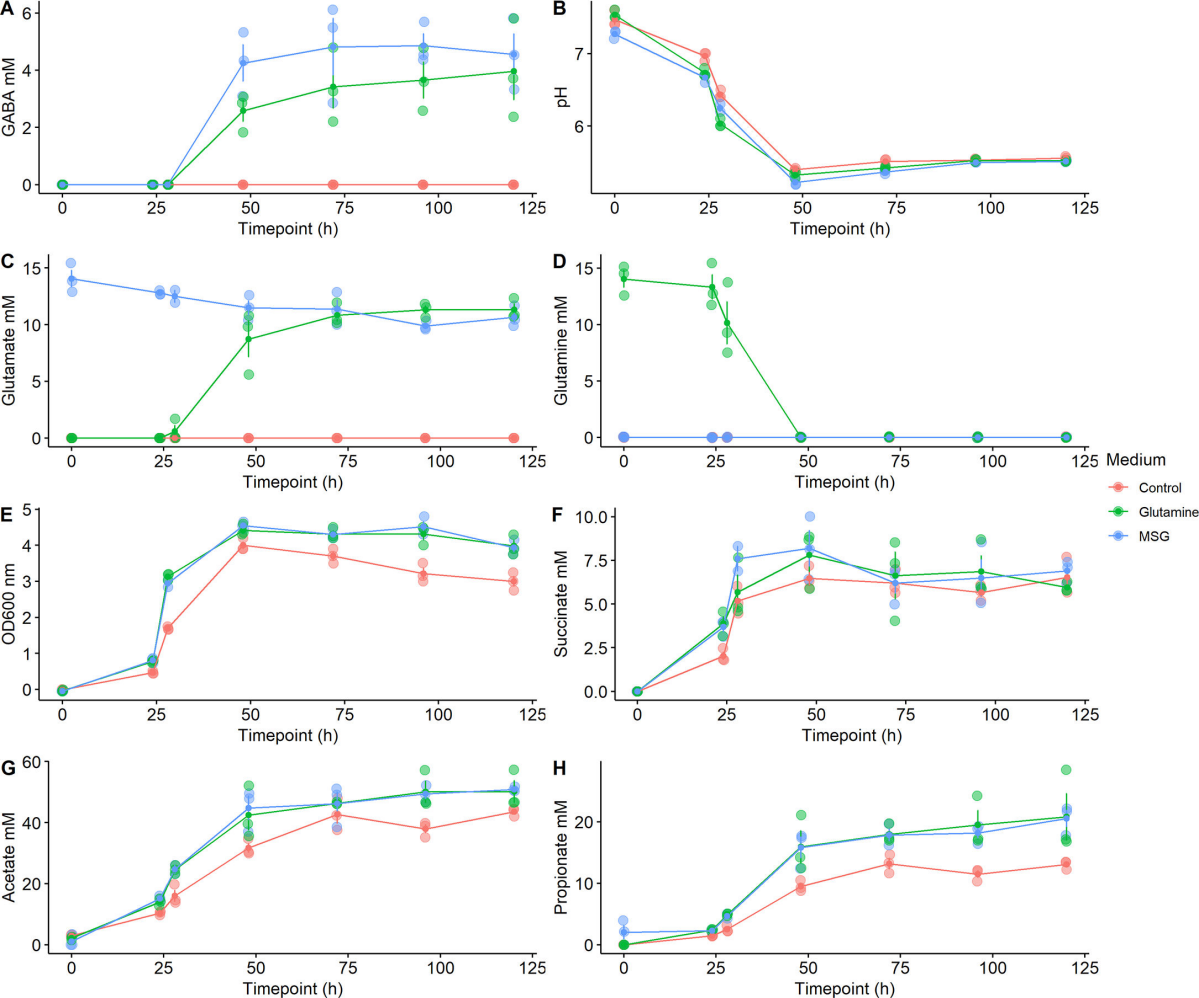
GABA production is related to the pH levels of the culture. *A. muciniphila* grew in basal media with N-acetylglucosamine with and without GABA precursors (either glutamate or monosodium glutamine). (**A**) Amount of GABA present in the cultures and (**B**) pH levels. Amount of (**C**) glutamate and (**D**) glutamine in the cultures. (**E**) Levels of optical density. Production of metabolites (**F**) succinate, (**G**) acetate, and (**H**) propionate.

GABA was first detected at 48 h (4.2 ± 0.77 mM, 28% conversion of the supplied precursor and 2.6 ± 0.5 mM, 17.3% conversion), when the pH of the medium dropped below pH 5.5 in the samples grown in the presence of glutamate or glutamine (Fig. 2A and B). Following this, GABA levels rose steadily to a final concentration of 4.6 ± 0.8 mM (30.7% conversion) for the glutamate-supplemented samples and to 4.0 ± 1.2 mM (26.7% conversion) for the samples with added glutamine (Fig. 2A). Together with the detection of extracellular GABA, pH levels reversed back to 5.5 at the last time points.

In samples with added glutamate, the amount of glutamate decreased progressively over time (Fig. 2C). Interestingly, in the samples with added glutamine, the amount of glutamate started increasing after 24 h, suggesting that *A. muciniphila* converts the available glutamine to glutamate, which coincides with the initial decrease in pH (Fig. 1B and C). This process then provides glutamate for GABA production (Fig. 2A through C).

Finally, we found that *A. muciniphila* grew to a higher optical density (OD_600_), and pH levels decreased faster when glutamate or glutamine were added in the medium, as compared to the control cultures (Fig. 2B through E). The decline in pH can be explained by the higher production of succinate, acetate, and propionate, which acidify the medium (Fig. 2F through H).

### *A. muciniphila* produces high amounts of GABA in pH-controlled bioreactors with low pH

To further study *A. muciniphila*’s production of GABA as a response to pH, experiments were continued in bioreactors operated in batch mode to tightly control pH levels. First, *A. muciniphila* was grown at pH 6.8 or 5.8 in basal medium supplemented with 100 mM N-acetylglucosamine (GlcNAc) and 30 mM glutamate. No GABA was produced after 125 h (Fig. S1).

**FIG 3:**
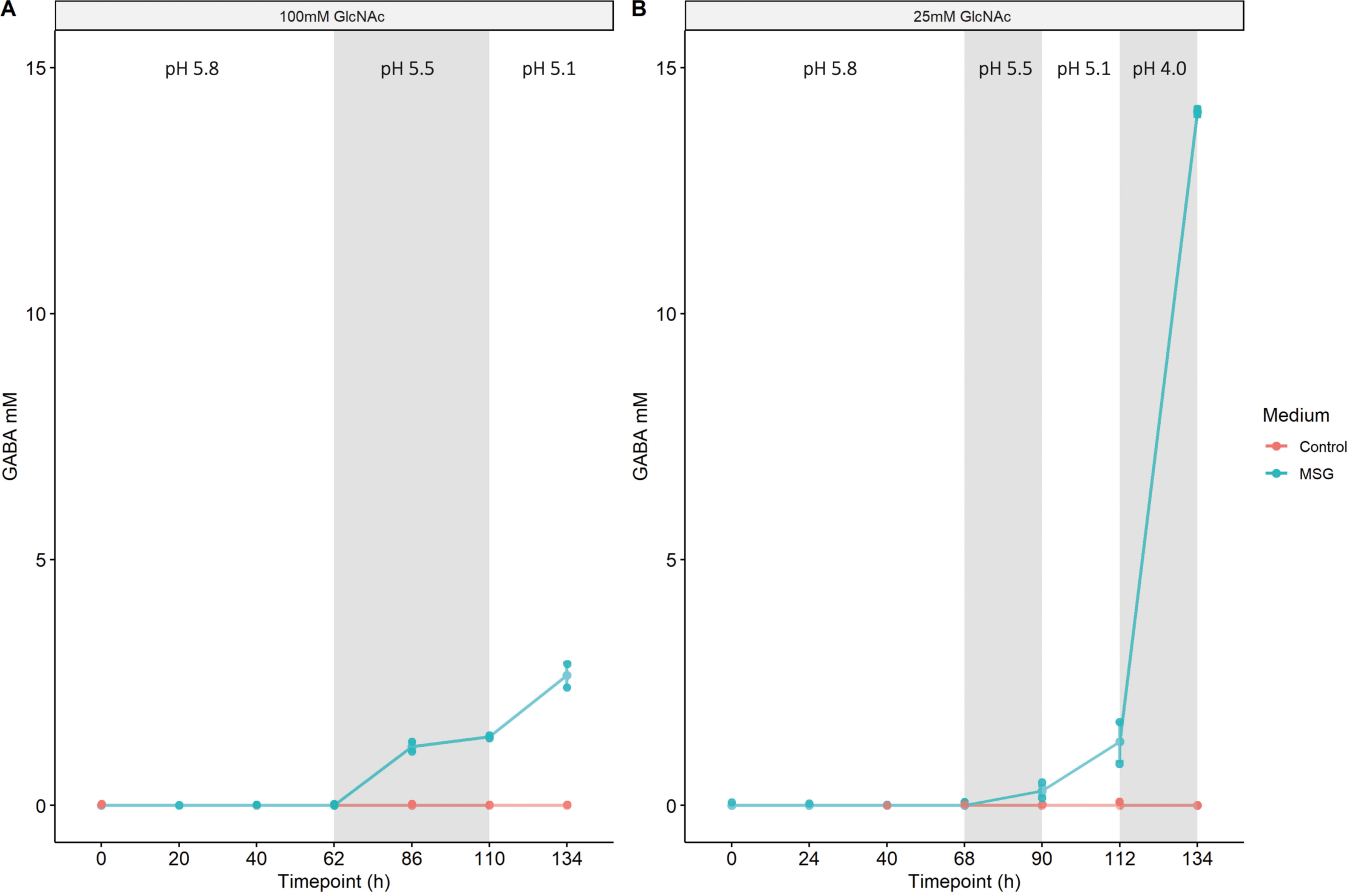
Production of GABA by *A. muciniphila* in reactors with controlled pH with (**A**) 100 mM GlcNAc and (**B**) 25 mM GlcNAc. Samples were grown either in the absence or presence of glutamate. White/gray areas indicate a change in pH, from left to right: pH 5.8, 5.5, 5.1, and (only in B) 4.0. Quantification was done with high-performance liquid chromatography tandem mass spectrometry, and measurements were performed in duplicate.

Next, the same experiment was repeated (basal medium supplemented with 100 mM GlcNAc in either the absence or presence of 30 mM glutamate) at pH 5.8 until cultures reached the stationary phase. Then, pH was adjusted to 5.5 and maintained for 48 h, after which pH was adjusted to 5.1 and maintained there for another 24 h. GABA was produced when the pH levels were 5.5 and lower, but only when glutamate was present in the medium. After 24 h at pH 5.5, GABA was first detected at a concentration of 1.2 ± 0.14 mM, and after 48 h, this increased slightly to 1.4 ± 0 mM. Then, after 24 h at pH 5.1, the amount of GABA increased to an average of 2.65 ± 0.35 mM (8.8% conversion) (Fig. 3A).

To verify that the concentration of carbon source is not related to GABA production, we repeated the experiment using 25 mM GlcNAc (Fig. 3B). This time, after the stationary phase was reached, the pH was adjusted in 24-h increments from 5.8 to 5.5, 5.1, and finally 4.0. Again, GABA was detected only when glutamate was present in the medium. After 24 h at pH 5.5, the GABA concentration was 0.3 ± 0.1 mM. After 24 h at pH 5.1, the amount of GABA was raised to an average of 1.3 ± 0.5 mM. This time, an extra step of pH 4.0 was included after pH 5.1. This led to an increase in GABA to an average of 14.1 ± 0.1 mM (47% conversion) after 24 h (Fig. 3B). No difference was observed between the levels of acetate and propionate between the samples with and without added glutamate (Fig. S2).

### *A. muciniphila* glutamate decarboxylase is expressed regardless of the presence of GABA precursors

To explore whether the expression of Amuc_0372, encoding GAD, is dependent on the presence of the GABA precursors glutamate and glutamine, we compared proteomic data between samples with and without glutamate at end-point measurements. Amuc_0372 was among the 30 most detected proteins, regardless of the presence of glutamate. We did not find a significant difference in the expression of Amuc_0372 between the GABA-producing cultures and the control cultures (Table 1). Next to that, we also looked at the other proteins required for GABA synthesis: Amuc_0038 (glutaminase) and Amuc_0037 (amino acid permease), and again, no difference in expression was found between the GABA-producing and control cultures (Table 1). Furthermore, we examined for the presence of 4-aminobutyrate aminotransferase and succinate-semialdehyde dehydrogenase, the two enzymes that can use GABA to produce succinate, but both of them were not detected. Finally, our analysis did not show a significant difference in any proteins after adjusting for multiple comparisons using the Benjamini-Hochberg procedure, meaning that there were no differentially expressed proteins between the GABA-producing and non-producing cultures (Fig. S3). This suggests that the GABA-producing pathway is expressed regardless of the presence of GABA precursors.

**TABLE 1:** Protein expression of GABA-related proteins from bioreactor samples at time point 120 h^*a*^

Locus_tag and name	Locus_tag	Average log2(LFQ) GABA producing	Average log2(LFQ) control	Fold change	Error *P*-value
Amino acid permease	Amuc_0037	28.397	27.875	−0.72	0.46
Glutaminase	Amuc_0038	29.878	29.329	−0.76	0.46
Glutamate decarboxylase	Amuc_0372	32.586	32.037	−0.84	0.43

^*a*^ All raw data can be found in the supplemental Excel file.

### *A. muciniphila* GAD is active between pH 6.0 and 4.0

**FIG 4:**
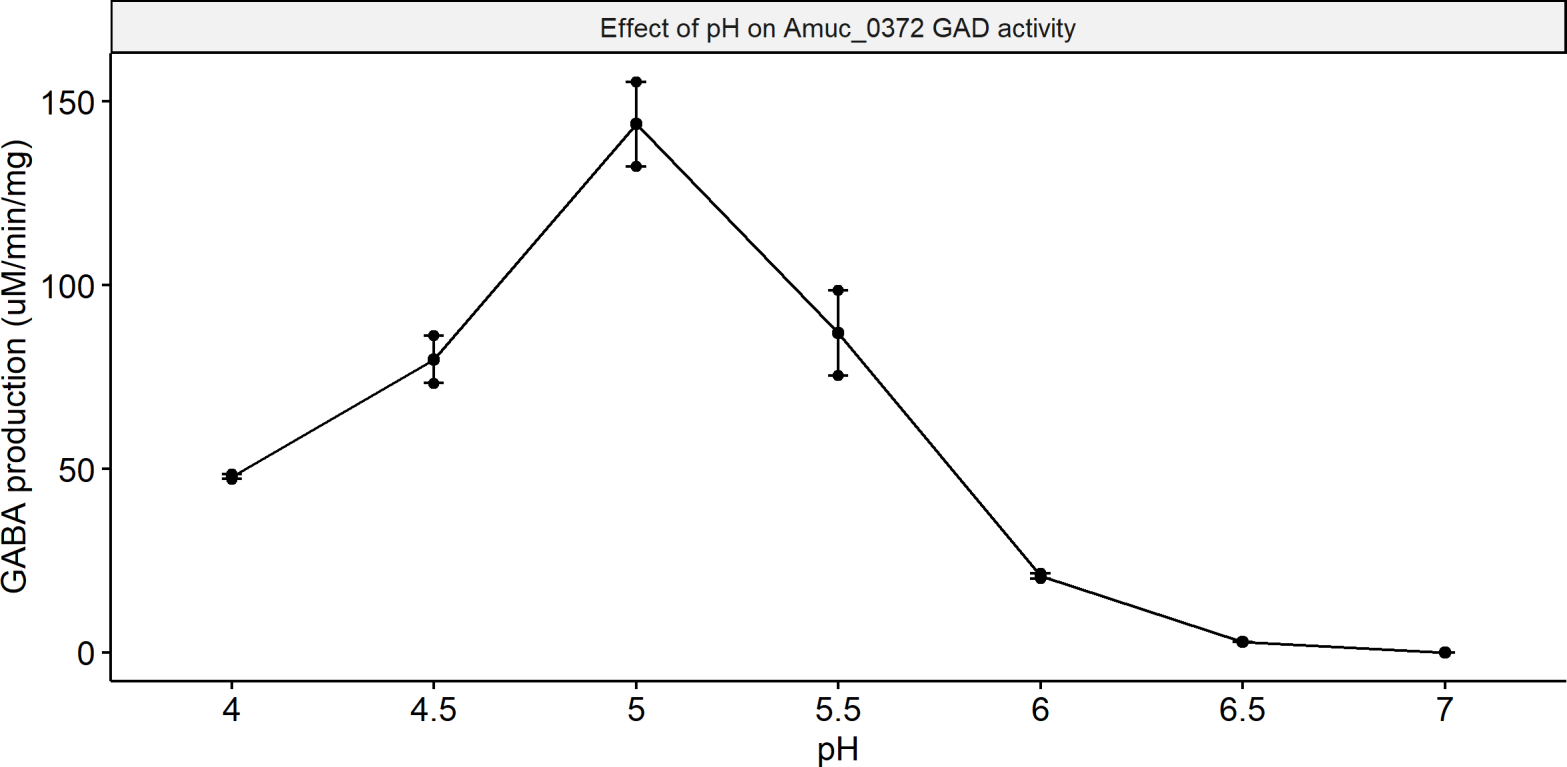
Effect of pH on the amount of GABA produced per minute per µg of GAD (Amuc_0372) from *A. muciniphila*. Activity was found between pH 4.0 and 6.0, with an optimum at pH 5.0. Enzymatic assays were performed in duplicate in the presence of the cofactor PLP.

To determine at which pH *A. muciniphila*’s GAD is active, we heterologously expressed Amuc_0372 in *E. coli*. The enzymatic assays using the purified protein showed Amuc_0372 activity between pH 6.0 and 4.0, with an optimum at pH 5.0 (Fig. 4). The protein did not show any activity at pH levels higher than 6.0, further supporting that the GAD response is triggered by acid stress. To exclude the possibility that native GAD proteins from *E. coli* were present and active after column purification, assays were performed using the cell-free extract from *E. coli* carrying an empty vector (i.e., without the Amuc_0372 gene) or carrying a vector containing and expressing Amuc_0372. No activity was detected for the strain carrying the empty plasmid, confirming that activity in our assays was attributed to the purified Amuc_0372 produced (Fig. S4).

## DISCUSSION

In the present study, we demonstrated the ability of *A. muciniphila*, a gut commensal mucin specialist and a potential next-generation probiotic bacterium, to produce GABA, the major inhibitory neurotransmitter of the mammalian central nervous system. We show that *A. muciniphila* produces GABA as a response to low pH when the GABA precursors glutamate or glutamine are present in the medium. It is likely that *A. muciniphila* can counter the effect of low pH in the medium through the production of GABA. Furthermore, through *in vitro* enzymatic assays, we verify that acidic pH is essential for the activation of GAD in *A. muciniphila*, as the optimal activity was at pH 5, and the enzyme is inactive at pH higher than 6.

Similar to our results, previous studies on human gut bacteria showed that GABA is produced as a response to low pH (19). *A. muciniphila* is able to grow in a pH range between 7.5 and 5.5 and is found to colonize the distal colon more than the proximal colon because of its higher pH (20). Although pH levels in the human gut are considered to range between 5.7 and 7.5 (21), there are studies that report pH levels around 5.5 and even close to 5 for some individuals in the colon (22, 23). Moreover, literature suggests that across the gut there are sections in the large intestine where pH levels are close to 5.5, such as in the proximal colon. This is mainly due to the active fermentation of dietary substrates, leading to significant concentrations ofshort chain fatty acids (24). This explains why it is important for *A. muciniphila* and other gut bacteria to have an acid-protective system, as it is required for essential to thrive in the gut (20, 25). In this case, low pH levels are assumed to be counteracted by the production of GABA, which consumes excess protons (Fig. 1). One proton is consumed when glutamate is the precursor, and two protons are consumed when the precursor is glutamine (Fig. 1).

Intracellular pH is generally considered stable, with even an extracellular decrease from pH 7.5 to 5.5 leading to a recovery of intracellular pH within minutes in *E. coli* (26). However, when high amounts of permeant acids, such as acetate, are present in the medium, the intracellular pH does not recover (26). Addition of acetate to the medium of *E. coli O157:H7* was also found to decrease the difference between the extracellular and intracellular pH from Δ0.9 to Δ0.2 (27). Assuming the relationship between *A. muciniphila* extracellular and intracellular pH works similarly to that of *E. coli*, production of GABA by *A. muciniphila* is possible to take place in sections of the gut with active fermentation, where acetate is present in high amounts since the extracellular and intracellular pH are almost the same. This is in line with our results from the pH-controlled bioreactors and the enzymatic assays, as the enzymatic activity of GAD was found to start at pH 6 (Fig. 4), and the GABA being produced in culture (Fig. 2 and 3) started at pH 5.5. As *A. muciniphila* produces high amounts of acetate, the difference between intracellular and extracellular pH could be in the same range as that of *E. coli*. However, pH measurements of *A. muciniphila* cytoplasm should be performed in the future to confirm this hypothesis.

Proteomic analyses showed no significant difference between GABA-producing and non-GABA-producing samples in terms of Amuc_0372 (GAD) production, implying that the protein is expressed regardless of the presence of the GABA precursors. Through our *in vitro* enzymatic assays, we showed that the optimal activity of *A. muciniphila* GAD protein is at pH 5. In *E. coli*, this pH-dependent activation of GABA is attributed to the structural changes that GAD undergoes under acidic conditions (28). Comparisons of the crystal structures of the GAD protein at pH 4.6 and 7.6 provided evidence for specific changes in three residues of the protein, which are essential for activation of the protein (28). Comparing the GAD gene and proteins from a selection of previously reported GABA-producing gut bacteria reveals that *E. coli* GAD and *A. muciniphila* GAD are genetically very close, with only *A. glycaniphila* GAD being closer to *A. muciniphila* (Fig. S6) (19). However, comparing residues shows that those responsible for changes in the *E. coli* GAD protein are not conserved in the *A. muciniphila* GAD (Fig. S7). Still, it is possible that the GAD protein from *A. muciniphila* undergoes structural changes, just as the *E. coli* GAD protein does, to explain the pH-dependent activity of the protein. However, protein structure analysis will be necessary to prove this notion.

Previous metagenomics-based studies showed that GAD is widely distributed among gut bacteria, but its activity and contribution to GABA production have been experimentally verified only in a few bacteria (5, 29, 30). Several of these bacteria, such as *E. coli* and *Bifidobacterium dentium*, can utilize GABA intracellularly for succinate production (31). However, similar to species within the *Bacteroides* genus, *A. muciniphila* lacks the succinate dehydrogenase gene that is reported to be essential for the production of succinate from GABA. This suggests that the produced GABA will likely not be used by the bacterium itself and instead be transported extracellularly, increasing GABA levels in the gut lumen. The bacteria-derived GABA in the intestine can be important for the ecology of the microbial communities in the human gut, as it can serve as a carbon and nitrogen source (19, 32). Next to that, bacteria-derived GABA is an immunomodulatory molecule, as it can affect various aspects of the immune system (33–35). Furthermore, changes in GABA-producing bacteria in microbial communities have been linked with depressive symptoms (19, 36, 37). These results suggest that bacteria-derived GABA not only might have an effect locally in the gut but might be also play a role in human mental health.

*A. muciniphila* is a commensal that is considered to be a next-generation probiotic bacterium that has been linked with several beneficial effects on the host, as it has been inversely correlated with obesity, diabetes, inflammation, and metabolic disorders. *A. muciniphila* has also been implicated in the gut-brain axis (38). A recent study used two different mouse models of epilepsy to show that a ketogenic diet confers anti-seizure effects (39). Despite the fact that ketogenic diets have been known for years as an effective treatment for epilepsy, their mechanism of action is not well understood (40). In the same study, the authors indicated a significant increase in the GABA/glutamate ratio in the brain hippocampus and also reported significant enrichment of *Akkermansia* spp. and *Parabacteroides* spp., in the animal feces. It is worth mentioning that *Parabacteroides* spp. have been previously reported to produce GABA, and here we demonstrate that *A. muciniphila* is also a GABA producer. Despite the consensus that peripheral GABA cannot cross the blood-brain barrier (BBB) (41) and that GABA found in the brain is exclusively produced within the brain, there are reports that peripheral GABA can cross the BBB in very small amounts (19, 42). Nevertheless, even if GABA cannot cross the BBB, it is possible that GABA has activity in the gut and that the two pools, brain and intestinal GABA, affect each other.

Overall, we show that *A. muciniphila* produces GABA in the presence of the GABA precursors glutamate and glutamine as a response to low pH. Our study demonstrates that to further understand the role of bacteria-derived GABA in the human gut, it is important not only to identify previously undescribed GABA-producing and -consuming bacteria but also to decipher the conditions under which GABA production is favorable for these bacteria. This knowledge is required for the design of supplements or dietary treatments targeting the enteric nervous system through GABA production by gut microbes or to stimulate the growth of specific gut bacteria that may be beneficial for the host.

## MATERIALS AND METHODS

### Bacterial culture and growth conditions for batch experiments

Batch cultures of *Akkermansia muciniphila* Muc^T^ (ATCC BAA-835, DSM 22959) were grown in basal media containing 100 mM GlcNAc as a carbon source and threonine (4 g/L) as the sole nitrogen source (43). To screen for GABA production, bacterial cultures were supplemented with 15 mM of the GABA precursors, monosodium glutamate or L-glutamine. Samples were inoculated with 1% (vol/vol) o/n preculture normalized to an OD of 1. Every 24 h, pH was measured for each culture, and bacterial growth was determined by measuring the optical density at 600 nm. Incubations were performed in serum bottles sealed with butyl rubber stoppers at 37°C under anaerobic conditions provided by a gas phase of 182 kPa (1.5 atm) N_2_/CO_2_.

### Growth conditions in bioreactors for batch experiments with controlled pH

To tightly regulate the pH of the medium, *A. muciniphila* was grown in batch bioreactors using the same basal medium as in the batch cultures, in either the presence or absence of 30 mM monosodium glutamate. For this, 1 L Eppendorf stirred-tank reactors on the DASGIP Bioblock system were used, which were set at 37°C and stirred at 50 rpm. Gassing was set at 80/20 N_2_/CO_2_ using the DASGIP MX4/4 gas mixing system, which was led through stainless steel diffusers with an outlet at the bottom of the reactors. The pH at inoculation was adjusted to 5.8 by the automatic addition of KOH and HCl. Samples were inoculated with 1% (vol/vol) o/n preculture normalized to an OD of 1. After 24 h in the stationary phase, the pH was readjusted to 5.5 for 24 h and then to 5.1 for another 24 h. In the final experiment, 25 mM instead of 100 mM GlcNAc was used, and after reaching the stationary phase, the pH was adjusted to 5.5 for 48 h, then 5.1 for 24 h, followed by a final adjustment to 4.0 for 24 h. Bacterial growth was determined for each bioreactor by OD_600_, and cells were checked using a microscope to rule out any changes in morphology.

### Metabolite quantification with high-performance liquid chromatography

The detection of organic acids acetate, propionate, and succinate was performed using high-performance liquid chromatography (HPLC) analysis as described previously (44). Samples were centrifuged for 5 min at full speed, and 800 µL of supernatant was transferred into HPLC vials containing 200 µL crotonate (10 mM in 0.1 N sulfuric acid) as an internal standard. The external standards were lactate, formate, acetate, propionate, butyrate, succinate, GlcNAc, and N-acetyl-galactosamine. Substrate conversion and product formation were measured with the Thermo Scientific Spectrasystem HPLC Shimadzu Prominence-1, LC2030C-Plus (Shimadzu Benelux, The Netherlands), equipped with an SH1821 column (Sugar SH1821, S/N F6378101, 300 × 8 mm, Shodex Showa Denko Europe). The column was kept at 75°C, and 0.01 N H_2_SO_4_ was used as eluent. The eluent had a flow rate of 1 mL/min, and metabolites were detected by determining the refractive index and identified by using standards of pure metabolites as described previously (44).

HPLC-ultraviolet-visible spectrophotometry (UV/VIS) was also used to determine the amounts of GABA, glutamate, and glutamine. Prior to analysis, amino acids were derivatized with dabsyl chloride. For this, a 10 µL sample, 100 µL 0.15 M NaHCO_3_ pH 9.0, and 200 µL freshly made dabsyl chloride (1.3 mg/mL in acetonitrile) were mixed and heated at 70°C for 20 min. After cooling down, 690 µL of 70% ethanol was added, and the sample was transferred into an HPLC vial. After derivatization, the measurement was performed on a Shimadzu Prominence-1, LC2030C-Plus_2_ELSD HPLC (Shimadzu Benelux), equipped with an Agilent Poroshell 120 EC-C18 column (250 × 4.6 mm, S/N 690970-902; Agilent Technologies Netherlands B.V.). The eluent was a gradient of acetonitrile (ACN), 100 mM formic acid (FA), and water. At the start, the ACN/FA/water ratio was 60/20/20% for 5 min. After 18 min, the amount of ACN/FA/water was linearly adjusted to a ratio of 22.5/7.5/70%, and after 22 min, the ratio was adjusted back to 60/20/20%. The flow was 1 mL/min, the column temperature was held at 40°C, and the injection volume was 5 µL. Detection was done with a UV/VIS detector at a wavelength of 436 nm.

### Confirmation and quantification of GABA with LC-MS/MS

After detection with HPLC-UV/VIS, GABA production was confirmed and quantified using HPLC-MS/MS. Sample analysis was carried out with a Nexera UPLC system (Shimadzu Corporation, Kyoto, Japan) coupled with an LCMS-8050 triple quadrupole mass spectrometer (Shimadzu Corporation). The UPLC unit consisted of an SIL-30AC autosampler, an LC-20ADXR solvent delivery module, a DGU-20ASR degassing unit, a CTO-20AC column oven, and an FCV-20AH_2_ valve unit. The samples (5 µL) were injected on a SeQuant ZIC HILIC 3.5 µm, 4.6 × 150 mm (Merck KGaS, 64271, Darmstadt, Germany) attached to a SeQuant ZIC HILIC PEEK coated guard column 20 × 2.1 mm (Merck KGaS, 64271, Darmstadt, Germany). The flow rate was set at 0.7 mL/min, and the column temperature was set at 40°C. The mobile phases consisted of 0.1% formic acid (solvent A) and acetonitrile with 0.1% formic acid (solvent B) with the following elution profile (*t* in [min]/[%B]): (0.0/90), (4.0/70), (10.0/20), (13.0/20), (15.0/90), and (18.0/90). MS/MS data were collected for 18 min. Positive ionization mode was used for the MS analysis. The voltage of the turbo ion-spray ionization was 4.0 kV. The temperatures of the electrospray ionization probe, desolvation line, and heat block were set at 300°C, 250°C, and 400°C, respectively. The pressure of the collision-induced dissociation gas was 4 kPa, whereas the flow rates of the drying gas, nebulizer gas, and heating gas were set at 10, 3, and 10 mL/min, respectively. The electrode voltage of Q1 pre bias (collision cell energy entrance potential), collision cell Q2 (collision energy, CE), Q3 pre bias (collision cell energy exit potential), parent, and fragment ion m/z of the multiple reaction monitoring transitions were optimized using support software (LabSolutions Shimadzu Corporation, Kyoto, Japan). For data on dwell time, Q1, CE, and Q3 bias, see Table S1. For GABA, the precursor ion was 104.0 m/z, and the product ions were 87.1 or 69.1 m/z. The most abundant fragment ion was selected for the quantitation of the analytes. The second fragment in ion yield was selected as structural confirmation based on the optimized SRM transition produced.

### Proteomic analyses

In total, 1 mL of *A. muciniphila* cultures was recovered from each bioreactor at time point 120 h and used for proteomic analysis. Cells were spun down by centrifugation for 10 min at max speed at 4°C in low-binding Eppendorf tubes. Pellets were washed twice with 200 µL of ice-cold 100 mM Tris buffer (pH 8). Cells were then resuspended in 50 µL of 100 mM Tris buffer (pH 8) and sonicated for 15 s. After sonication, protein concentration was measured, and proteins were reduced and digested as described for the second and third sets of proteomes in Feng et al. (45). Then, samples were cleaned using microcolumns that were prepared by cutting 2 C18 Empore disks and transferring them into a 200 µL tip. Subsequently, 200 µL of methanol was added to the tip, followed by 4 µL of 50% LichroprepC18 in ethanol slurry. Columns were washed with 100 µL of methanol, followed by equilibration with 100 µL of 0.1% formic acid in water. Samples with cell lysate were then added to the column and washed with 100 µL of 0.1% formic acid in water. Peptides were then eluted by adding 50 µL of formic acid-acetonitrile (1:1). Samples were then stored at −20°C until further processing with an nLC1000-Orbitrap Exploris480.

Data analysis for protein label-free quantification (LFQ) was performed as previously described (45). The database used for the analysis of the samples was the Akkermansia_muciniphila_baa-835_UP000001031 UniProt database. LFQ counts from each sample were first log2-transformed and normalized by slope. Fold change was calculated by subtracting the means of the normalized controls from those of the GABA-producing samples. *P-*value was determined using a two-sample equal variance *t*-test (46). Finally, the *P*-values were adjusted for multiple comparisons using the Benjamini-Hochberg procedure. A volcano plot was made using Perseus_1.6.2.1. First, contaminants as detected by maxQuant and proteins that were only detected once were removed. Then, −log LFQ values were plotted against −log *P*-values for each protein.

### Amuc_0372 cloning, expression, and purification

Amuc_0372 was amplified from the genomic DNA of *Akkermansia muciniphila* Muc (ATCC BAA-835, DSM 22959) with the Amuc_0372_Fw and Amuc_0372_Rv primers (Table 2), using the Q5 High-Fidelity DNA Polymerase (NEB) and by following the manufacturer’s instructions. The forward primer contained a 5′ extension to mediate Gibson assembly. The reverse primer contained a 5′ extension that introduced a histidine hexapeptide to the C-terminus of Amuc_0372 (necessary for purification; see below), along with a 5′ extension that is necessary for Gibson assembly. The pET-26b(+) vector was used for expression of Amuc_0372 and was linearized through PCR using the BG11571 and BG11572 primers (Table 2). NEBuilder HiFi DNA Assembly (NEB) was used to assemble the Amuc_0372 amplicon with the linearized pET-26b(+) vector, resulting in the pAmuc_0372_6His plasmid. pAmuc_0372_6His was introduced into chemically competent *Escherichia coli* BL21(DE3) cells, following the manufacturer’s instructions (NEB). Transformed *E. coli* BL21(DE3) cells were selected on solid Luria-Bertani medium (10 g/L tryptone, 5 g/L yeast extract, 10 g/L NaCl) containing 50 mg/L kanamycin. Successful cloning of pAmuc_0372_6His was confirmed through Sanger sequencing (Macrogen).

**TABLE 2:** Primers used in the cloning of Amuc_0372

Primer	Sequence
Amuc_0372_Fw	AAGAAGGAGATATACATATGTTTGATCCAAATCAAAAACCCG
Amuc_0372_Rv	GTTAGCAGCCGGATCTCAGTGGTGGTGGTGGTGGTGCTCGAGGGAATGGTGGAAAGCCGT
BG11571	GATCCGGCTGCTAACAAAG
BG11572	CATATGTATATCTCCTTCTTAAAGTTAAACAAAATTATTTC

To purify Amuc_0372, we used established purification protocols (47, 48). Briefly, *E. coli* BL21(DE3) bearing pAmuc_0372_6His was grown in 500 mL of liquid LB medium containing 100 mg/L of kanamycin and incubated at 37°C and shaking at 120 rpm until the OD_600_ reached 0.5–0.6. The cultures were then chilled on ice for 15 min and then induced with 0.2 mM isopropyl β- d-1-thiogalactopyranoside. The cultures were incubated for another 18 h at 18°C, shaking at 120 rpm. The cells were then harvested by centrifugation at 6,000 × *g* for 15 min at 4°C. Subsequently, the cell pellet was washed with 50 mL of 50 mM potassium phosphate buffer (KPi, pH 7.5) and then centrifuged at 6,000 × *g* for 15 min at 4°C. The resulting cell pellet was stored at −20°C until use.

For cell lysis, 20 mL of HA buffer (50 mM KPi, 300 mM NaCl, pH 8.0, and 20 mM imidazole) was used to resuspend the cell pellet. Two mini tablets of cOmplete protease inhibitor (MERCK) were also added and dissolved completely in the cell suspension. The cells were disrupted with sonication using a VS 70T tip (Bandelin SONOPLUS HD) and the following setup: 25% amplitude, 10 min total time, and 1 s on/2 s off. The cell lysate was centrifuged at 30,000 × *g* for 45 min at 4°C, and the supernatant was filtered through a 0.22 µm membrane filter (MDI Membrane Technologies).

The filtered supernatant was used for protein purification using an ÄKTA go Protein Purification System (GE Healthcare Life Sciences). The first step included loading the filtered supernatant on a 5 mL His Trap HP column (GE Healthcare Life Sciences), which was equilibrated with HA buffer. The bound proteins were eluted from the column using the HB buffer (50 mM KPi, 300 mM NaCl, 500 mM imidazole, pH 8.0). The fractions that contained the eluted proteins were collected, combined, and diluted five times with AA buffer (50 mM KPi, pH 7.0). To analyze the purity of the obtained protein, the fractions were loaded on a 10% Mini-PROTEAN TGX precast gel following the manufacturer’s instructions (Bio-Rad). Protein purity was checked on gel, and fractions containing the highest amount were used in downstream experiments (Fig. S5). Finally, the protein content of the samples was measured using Qbit.

### GAD enzyme assay

The GAD activity assay was conducted as follows: the reaction mixture consisted of 396 µL of 50 mM sodium citrate with 0.1 mM PLP and 10 mM L-glutamate and 4 µL of the purified enzyme solution. After incubation at 37°C for 20 min, the reaction was stopped by placing the sample vials in boiling water for 10 min. The generated GABA was measured by HPLC-UV/VIS as described above. All enzymatic assays were carried out in duplicate.

### Phylogenetic analysis

The phylogenetic tree showing the presence or absence of GAD in human gut bacteria was made using Interpro functional predictions from the publicly available MGnify species catalog (49). To visualize these results, the species were annotated using the R package ggtree (15).

Clustal Omega was used for multiple alignments of the GAD genes and proteins. Phylogenetic trees were visualized using the publicly available Interactive Tree Of Life program. The selection of bacteria was based on previously published data (19).

## SUPPLEMENTAL MATERIAL

Figure S1 aem.01121-23-s0001.tif No GABA detected in bioreactors at ph 5.8 and 6.8.

Captions of supplemental figures aem.01121-23-s0002.docx Fig. S1 to S7 captions and Table S1.

Figure S2 aem.01121-23-s0003.tif OD, acetate, and propionate production.

Figure S3 aem.01121-23-s0004.tif Volcano plot proteomics.

Figure S4 aem.01121-23-s0005.tif GABA production in enzymatic assays.

Figure S5 aem.01121-23-s0006.eps SDS gel of purified HIs-tagger amuc_0372.

Figure S6 aem.01121-23-s0007.eps Phylogenetic tree comparing *gad* genes and proteins.

Figure S7 aem.01121-23-s0008.tif Protein alignment between *E. coli* and *A. muciniphila*.

Supplemental GAD gene dataset aem.01121-23-s0009.xlsx Excel showing presence or absence of GAD gene in gut bacteria.

Supplemental proteomic dataset aem.01121-23-s0010.xlsx Supplemental Excel file containing proteomic data.

ASM does not own the copyrights to Supplemental Material that may be linked to, or accessed through, an article. The authors have granted ASM a non-exclusive, world-wide license to publish the Supplemental Material files. Please contact the corresponding author directly for reuse.
