# Supplementary figures and images for: Physiology of γ-aminobutyric acid production by Akkermansia muciniphila
Source: Appl Environ Microbiol. 2023 Dec 13;90(1):e01121-23. doi: 10.1128/aem.01121-23 (PMC10807452; doi:10.1128/aem.01121-23)

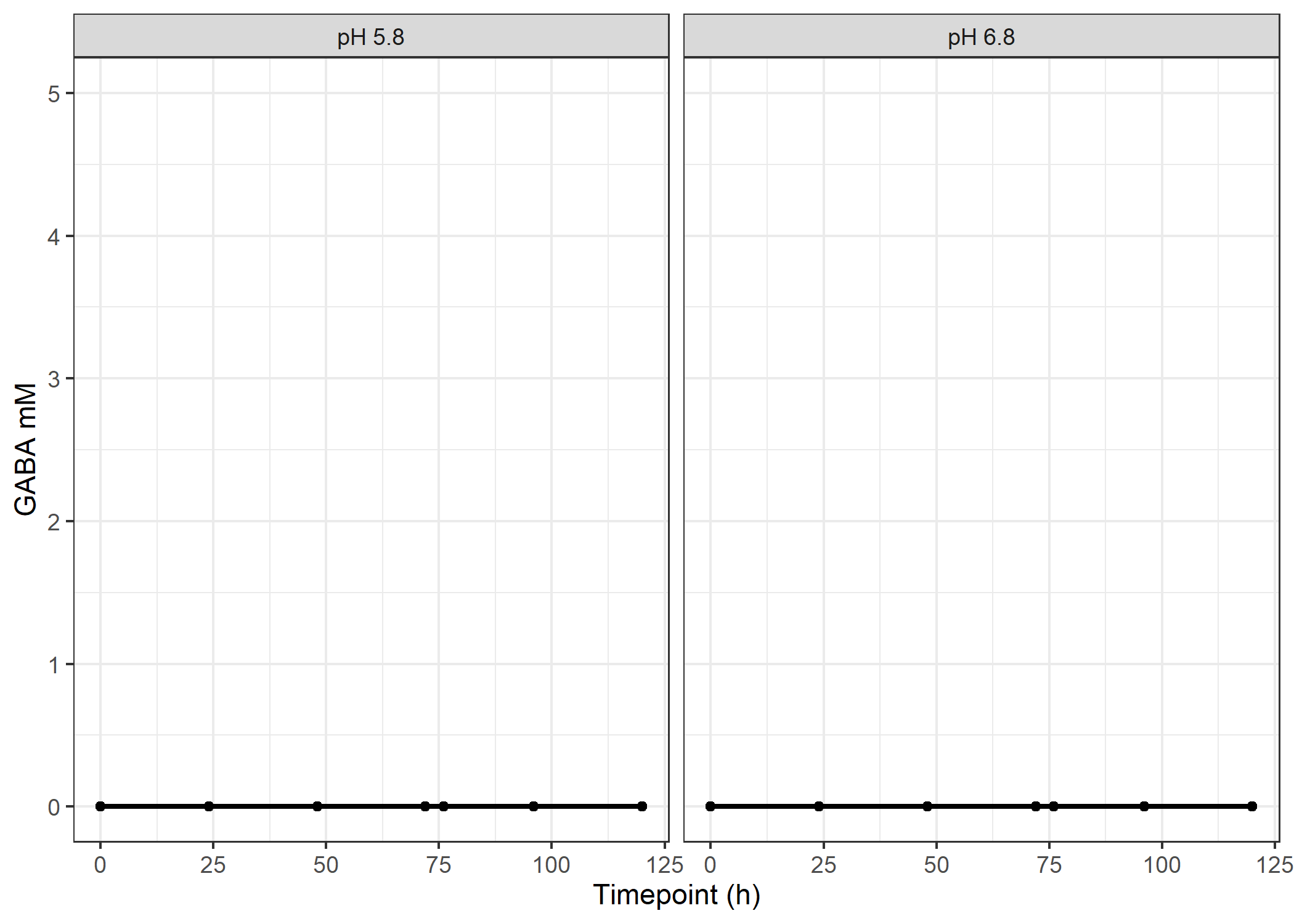

Supplement: Figure S1 — No GABA detected in bioreactors at ph 5.8 and 6.8. [file aem.01121-23-s0001.tif]

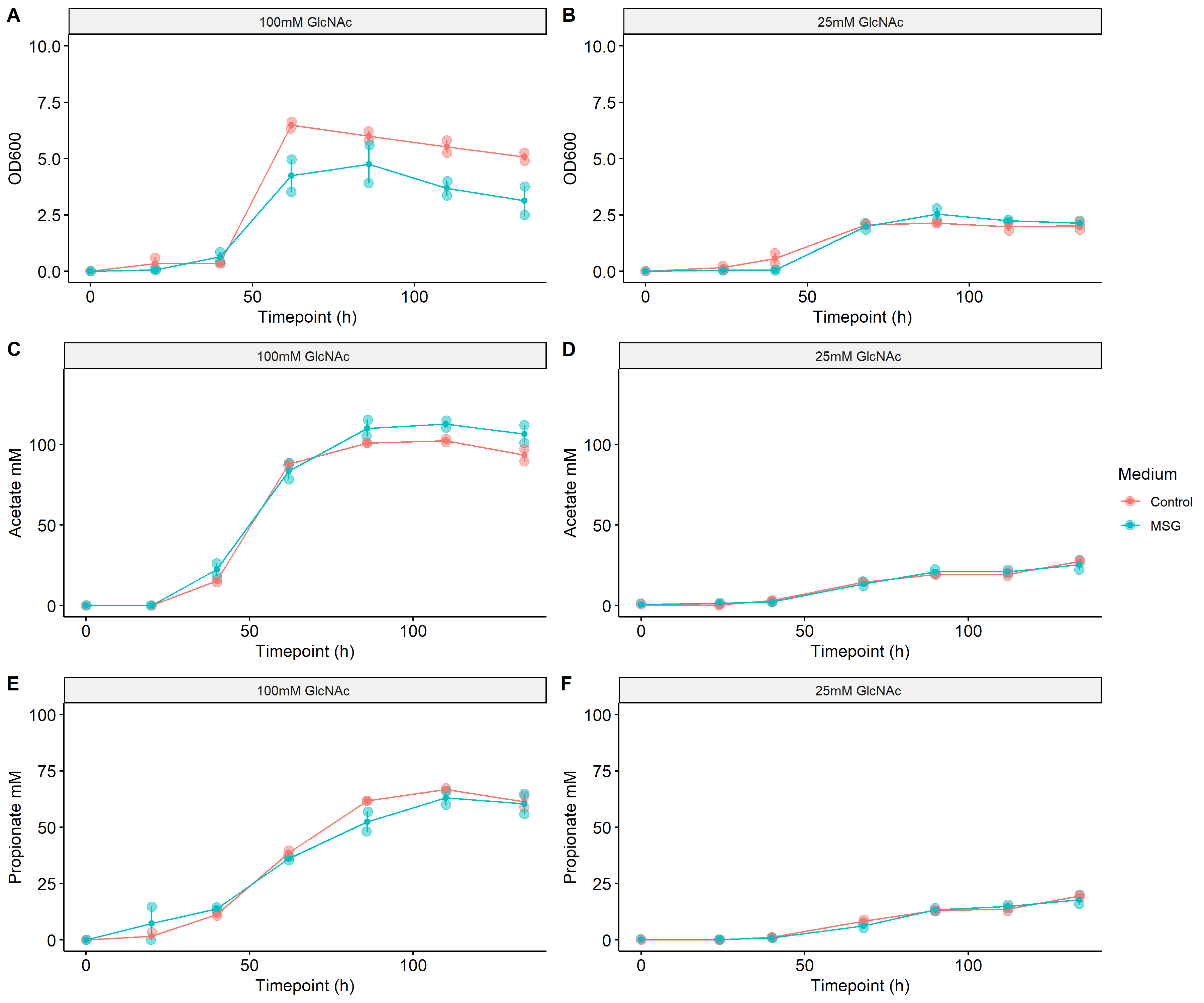

Supplement: Figure S2 — OD, acetate, and propionate production. [file aem.01121-23-s0003.tif]

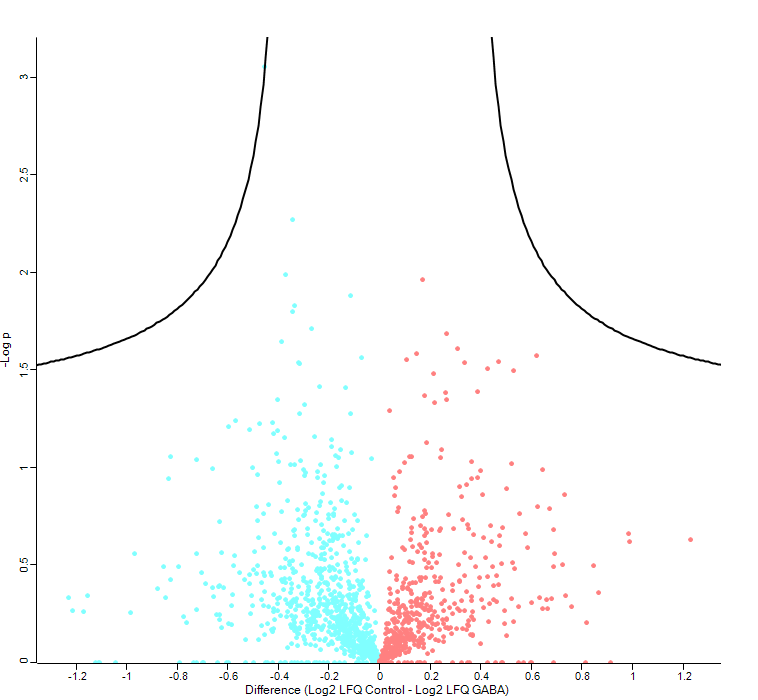

Supplement: Figure S3 — Volcano plot proteomics. [file aem.01121-23-s0004.tif]

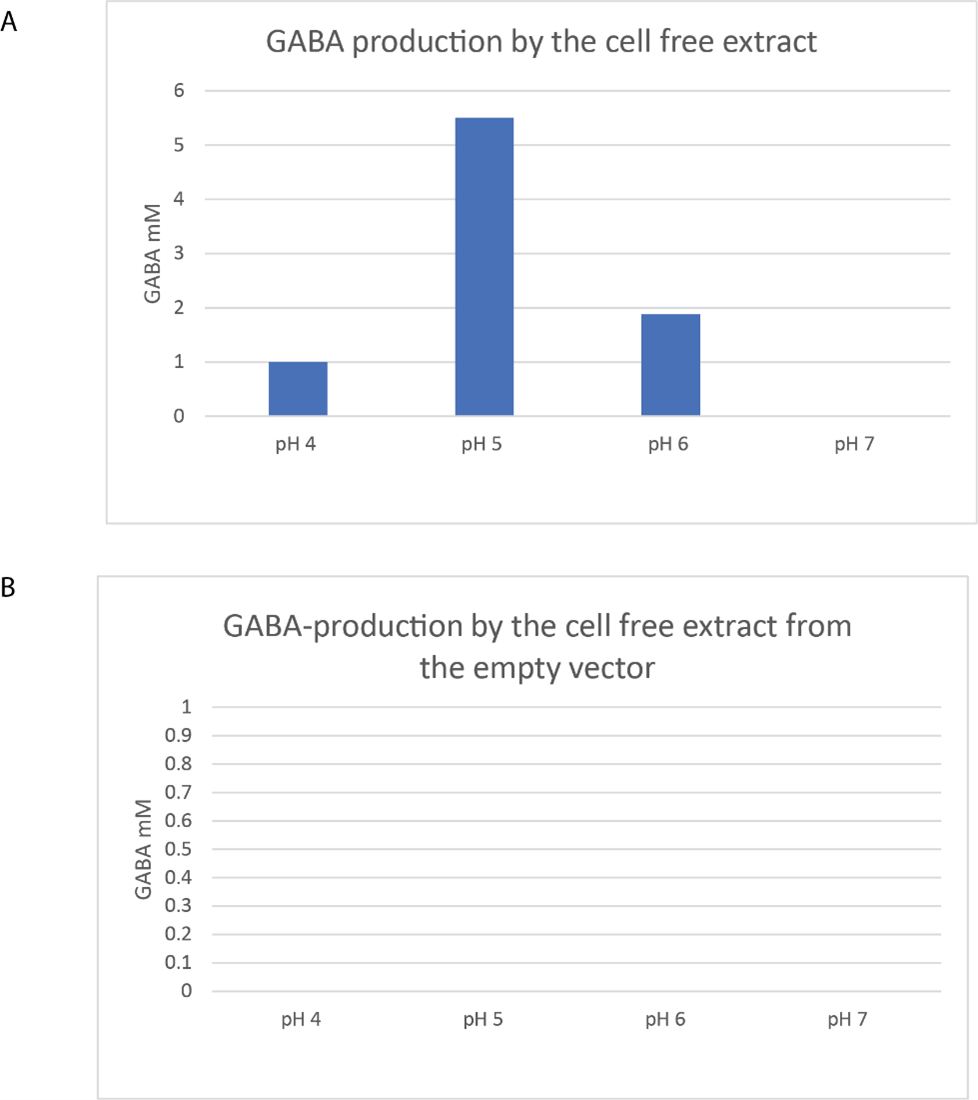

Supplement: Figure S4 — GABA production in enzymatic assays. [file aem.01121-23-s0005.tif]

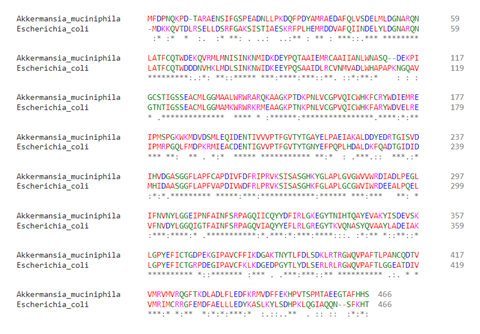

Supplement: Figure S7 — Protein alignment between E. coli and A. muciniphila. [file aem.01121-23-s0008.tif]
