# Supplementary material for: Physiology of γ-aminobutyric acid production by Akkermansia muciniphila
Source: Appl Environ Microbiol. 2023 Dec 13;90(1):e01121-23. doi: 10.1128/aem.01121-23 (PMC10807452; doi:10.1128/aem.01121-23)
Supplement: Captions of supplemental figures — Fig. S1 to S7 captions and Table S1. [file aem.01121-23-s0002.docx]

## Supplemental Figure Captions & Tables

**Supplementary Figure 1.** No GABA was detected when bioreactors were operated at pH levels 5.8 and 6.8 despite the presence of 30 mM of monosodium glutamate (MSG) in the cultivation medium.

**Supplementary Figure 2.** Optical density (A,B) and the production of acetate (C,D) and propionate (E,F) in mM, during the experiments with 100 mM or 25 mM of GlcNAc as carbon source. Measurements are in duplicates.

**Supplementary Figure 3.** Volcano plot of detected proteins. No statistically significant differences were found (points above the black line) between the GABA producing, and control samples.

**Supplementary Figure 4.** Amount of GABA produced during enzymatic assays with cell free extracts from A) the Amuc_0372 expressing *E. coli* and B) the *E. coli* carrying an empty vector after 20 minutes at 37°C.

**Supplementary Figure 5.** SDS gel of purified His-tagged Amuc_0372 (GAD) from *A. muciniphila* per fraction. Fractions 1.9 and 1.10 were used for the analysis because of their high amount of protein and purity after his-tag. Protein content of Fractions 1.9 and 1.10 was 1060 and 792ug/µL respectively.

**Supplementary Figure 6.** Phylogenetic tree comparing the GAD genes (A) and proteins (B) from different gut bacteria. *A. muciniphila* GAD is evolutionary closest to *Akkermansia glycaniphila*, followed by *Escherichia coli* GAD. Trees were constructed with ClustalOmega using default settings.

**Supplementary figure 7.** Protein alignment and conserved residues between glutamate decarboxylase of *E. coli* and *A. muciniphila* GAD. Asterisks (*) denote conserved regions, colons (:) represent a conservative mutation and points (.) represent semi-conservative mutations.

**Supplementary table 1.** Precursor ion, second fragmentation, dwell time, Q1, CE ad Q3 pre bias as selected for LC-MSMS of GABA.

| Metabolite | Precursor ion (m/z) | Product ion (m/z) | Dwell time (ms) | Q1 Pre Bias (V) | CE (V) | Q3 Pre Bias (V) |
| --- | --- | --- | --- | --- | --- | --- |
| GABA | 104.0000 | 87.1000 | 32 | -12.0 | -14.0 | -16.0 |
|  | 104.0000 | 69.1000 | 32 | -13.0 | -17.0 | -12.0 |
